# Measuring economic consequences of preterm birth - Methodological recommendations for the evaluation of personal burden on children and their caregivers

**DOI:** 10.1186/2191-1991-1-6

**Published:** 2011-07-20

**Authors:** Jan-Marc Hodek, J-Matthias von der Schulenburg, Thomas Mittendorf

**Affiliations:** 1 University of Bielefeld, Department of Health Economics and Health Care Management, Bielefeld, Germany; 2 Leibniz University Hannover, Center for Health Economics, Hannover, Germany; 3 herescon gmbh, Hannover, Germany

**Keywords:** Preterm birth, Low birth weight, Burden of disease, Parents, Out-of-pocket expenditures, Quality of life, Emotional distress

## Abstract

This study aims to identify the impact of a preterm birth on financial and emotional burden from the families' perspective. Additionally, a comprehensive schedule of recommendations for a sufficient evaluation of all aspects of burden is developed. Based on the results of a literature search relevant categories and sub-domains for a questionnaire covering multiple aspects of associated financial and emotional burden are identified and converted into a recommendation scheme. Results of the literature search illustrate the large extend of burden of prematurity on parents. This results in substantial out-of-pocket expenditures (OOPE) and emotional distress to the parents besides the medical problems and further financial costs to the health insurance system. According to the results on infants' state of health, OOPE and emotional distress are significantly increased with decreasing gestational age. OOPE for transportation often amounts to the main parental cost dimension. Moreover there is some evidence for a high magnitude of reduced income and missed work days. The family perspective has to be taken into account when calculating the overall costs of preterm births from a societal point of view. However, in recent years economic evaluations were performed rather inhomogeneously in this field. For future studies a) direct medical costs, b) direct non-medical costs, c) indirect costs as well as d) intangible costs (in terms of emotional distress and reduced quality of life for caregivers and children) are the main categories that should be evaluated measuring personal burden of preterm birth on families adequately. A detailed list of specific sub-domains is given. Additionally, the recommendations are not restricted to application in infants born preterm and/or at low birth weight.

## Introduction

Although the number of births in most industrialized countries remained relatively stable in recent years, an increasing incidence of infants with low gestational age (≤36 weeks of gestation) and low birth weight (<2,500 g) can be observed. For example, in Germany the proportion of infants with low birth weight rose by nearly a fourth from the 1980 level. Increasing maternal age and fertility treatments in many western countries are only two of several possible reasons for this development. For example, in Germany the percentage of infants born preterm is about 9-10% of all newborn children, which comes to approximately 60,000 infants per year. About 1-2% is even born before the 32^nd ^week of gestation. Regarding the entirety of all newborns, 6,8% are weighing less than 2,500 g and 1.2% less than 1,500 g (8,090 infants in 2005). Similar trends can be observed in other industrialized countries [1-3].

Those children who do survive have a higher risk of future health-related and developmental problems ranging from severe motor and sensory impairments to attention deficit disorders (ADHD) and learning difficulties. Clinical complications may include chronic lung disease, acute respiratory and/or gastrointestinal prob-lems as well as visual impairments or severe infections [4-7]. On the other hand dramatic advances in neonatal care and perinatal practices have resulted in increased survival chances of low gestational age and birth weight infants. Together these effects increase the cost of care provided to these children during the neonatal period and in later periods of life as well.

Recently the Institute of Medicine (IOM) in the US has prepared a comprehensive report called "Preterm birth, causes, consequences, and prevention" describing prematurity as an important public health issue. In the US this was elevated to the government and the "Preemie Act" signed in December 2006 decreed an expan-sion of research related to care, treatment and outcomes of preterm birth and low birth weight infants as well as public and provider education. A fundamental recommendation of the IOM report was to increase efforts aimed at understanding all aspects in the provision and perception of health care related to preterm birth. One of the issues envisaged is the burden posed by preterm infants on parents and families [4]. Moreover, in a recent study van Exel et al. emphasized that the overall healthcare sector strongly depends on informal care provided by families and other caregivers. According to their analyses informal caregivers may experience significant burden as well as health and well-being effects. Resource allocation decisions should always account for these "invisible-hands"-effects in the social environment of patients [8].

So far, existing research on costs of prematurity primarily focused on the (high) costs for initial hospitalization and associated neonatal intensive care for preterm infants from a health insurance perspective. In contrast, only little is known about the magnitude of the public burden beyond this early hospitalization (re-hospitalization, outpatient services, and medication) and non-medical costs like expenses for special education or indirect costs because of lost productivity, especially from the families' perspective. Aim of this paper is therefore to explore this issue further by reviewing existing literature in order to describe the financial and emotional burden of prematurity on parents. Additionally this study aims to develop recommendations on how to measure familial burden of disease in future studies.

## Methods

Based on a literature search, the objective is to gather a deeper insight into the medical and associated financial (out-of-pocket expenditures - OOPE) and emotional burden (quality of life - QoL) of a preterm birth and/or low birth weight on families. For this first part, a narrative literature search was conducted covering the time until April 2009. Main objective of this first part is not a complete evaluation or classification of all relevant studies, but to identify and to analyse different methods to quantify burden on parents. The following databases and search engines were used: MEDLINE, German Medical Science, Karger, Kluwer, Thieme and Springer bibliographic databases as well as Scirus search engine. The keywords used for the search were: (preterm OR premature OR low birth weight) AND (parent OR caregiver OR mother OR father) AND (cost OR out of pocket expenditure OR OOPE OR out of pocket payment OR OOPP OR quality of life OR burden of disease). Relevant publications needed to analyze at least one category of burden on parents or other caregivers and not only the perspective of the health care system. Because of expected scarceness of studies other inclusion or exclusion criteria were not defined. All different topics or cost domains that are identified through that process will be classi-fied and listed in structured table 1.

**Table 1:** Applicable domains to measure burden of disease: direct medical costs, direct non-medical costs, indirect costs, and quality of life aspects

Resource utilization domain	Possible contents and practical issues
***Health care co-payments or OOPE*** ***(direct medical costs)***	

*Outpatient*	*Co-payments or deductibles; health care services not covered by health* *plans and paid for by the parents*

- visits to physicians (general practitioners and specialists)	Additional services like German "IgeL"; (follow-up) visits causing a surgery fee or other co-payments

- visits to non-physicians	E.g. physiotherapy, ergotherapy, logopedics, osteopathy, massages, animal/music therapy, psychotherapy etc. (co-)paid for by the parents

- medication	Parental drug expenses (OTC drugs not covered by the insurance plan or co-payment for Rx)

- aids and devices	E.g. inhalators, home monitor, glasses, orthotics, wheelchair, specialized pushchair, sitting aid, hearing aid, corset etc. (co-)paid for by the parents

- outpatient/home care	Nursing staff, specialized bed (co-)paid for by the parents

	

*Inpatient*	*Co-payments or deductibles; health care services not covered by health* *plans and paid for by the patient*

- initial hospitalization	Co-Payments/deductibles for extra therapies/services

- re-hospitalization	Co-Payments/deductibles for extra therapies/services

- rehabilitation/regimen	Co-Payments/deductibles for extra therapies/services

	

***Other disease-related OOPE*** ***(direct non-medical costs)***	

- transportation	Travel costs for hospital visits (initial hospitalization, re-hospitalization) and transport to therapies/specialists, including parking

- accommodation	Lodging costs during the infants' hospital stays

- home or car remodeling	Adaptations to the families' home or car

- meals	Physician-ordered food

- other/special medical approaches	Alternative therapies: naturopathy, homeopathy, light therapy etc. (possibly overlapping with visits to non-physicians, see above)

- childcare/babysitting for other siblings	During absence of parents while accompanying the preterm child to hospital visits or therapies

- special education/schooling	Coaching/tutoring (not relevant for infants, but in later years)

- home help	For housekeeping as parent time is required caring for the preterm child

- higher insurance premiums	In private health insurance or supplementary insurance

	

***Indirect costs***	

- income losses	Due to change in work status of parents; lost wages (in very later life this is relevant for the preterm child as well: indirect costs caused by future limited ability to work)

- missed working days	Does not automatically mean reduced income, but often absence causes problems at work (psychologically and perhaps financially in the long run as well)

- time losses (opportunity costs)	For care, travelling, hospital visits (asking how much of this time would otherwise have been spent to work)

	

***Intangible costs:*** ***Quality of life aspects***	

- QoL of children	Development problems, infections, disabilities with influence on physical, emotional and social functioning

- QoL/physical and emotional burden on parents or other caregivers	Prenatal phase (anxiety, self-reproaches), perinatal phase (stress related to birth, separation from baby on NICU), postnatal phase (psychological distress: fear of losing child/infections/development problems, self-reproaches, burden on relationship to siblings, marital stress, maternal depression, restricted social contacts, feeling of isolation etc.)

Based on the findings, the results of the review are used to complete a comprehensive list of recommendations for a sufficient evaluation of all burdens of preterm births on families concerned. The particular choice of which costs to include always depends on the respective perspective of a study: From the perspective of a family a) medical direct costs, b) non-medical direct cost, c) indirect cost and d) intangible costs (in terms of QoL aspects) are four the major categories, which have to be filled with information. The major categories were defined a priori. They were derived from general standards/basics of health economic evaluation and cover all possible types of burden. The main objective is to fill these four heading with special sub-domains covering all relevant aspects of personal burden and simultaneously to avoid double counting which is achieved by the analyses of the literature.

## Results

### Financial aspects

Valuing the economic burden of prematurity to society, it is very important to understand the full extent of costs as different cost domains are affected. However, it is accepted that these conditions impose a substantial financial burden not only on the health insurance, but on families and caregivers of the infants. There are several different cost categories that might be of importance.

A recent report by the IOM estimated that the societal economic burden associated with pre-maturity (≤36 weeks of gestation) in the US was at least 26.2 billion USD annually in 2005, or 51,600 USD per infant born preterm. These costs capture the annual discounted value of resources consumed per year in excess of what is projected to be used by infants born at term. Nearly two-thirds of these costs (33,200 USD) were ac-counted for by medical care services, with more than 85% of those delivered during early childhood (0-5 years). Maternal delivery costs accounted for an additional 3,800 USD per infant, early intervention service costs (for programs on the emotional, physical, and developmental outcomes, e.g. interventions for speech and language acquisition in very young children up to 3 years of age) contributed 1,200 USD annually and special educational services associated with a higher prevalence of disabling conditions among premature infants added 2,200 USD per person. Finally indirect costs, in terms of future lost productivity in the household and the labor force associated with disabling conditions of the children, contributed 11,200 USD per every preterm child. These cost components do not include costs of the caregivers for individuals with disabilities like out-of-pocket payments for education or loss of earnings during childhood which would have to be added [4].

Additional studies confirm an inverse relationship of neonatal and post-discharge costs with birth weight and gestational age [9-11]. According to the results of a comparable study for the European context the average overall 2-year-costs are 104,635 EUR for surviving infants born preterm (< 1000 g), compared to 3,135 EUR for normal birth weight children in Finland. Initial hospital costs alone accounted for 64% of total costs, whereas costs during the first and second year accounted for 20% and 13%, respectively [10].

However, evidence on financial burden (OOPE) is very limited. Costs during the neonatal period (mainly for initial hospitalization and associated OOPE, e.g. for transportation, child care or accommodation) can be distinguished from long-term costs after this period (co- and out-of-pocket payments for re-hospitalization, outpatient visits, pharmaceuticals, medical aids as well as non-medical costs for transportation, special education or time and earning losses).

#### Costs during the neonatal period

Several studies found an inverse relationship between gestational age or birth weight and hospital service costs during the neonatal period (initial hospitalization). Moreover they also showed that neonatal costs tended to be higher for preterm infants who survive compared with those who die. Furthermore hospital service costs during this period are highly related to the degree of surgical intervention performed on the infant and the level of assisted ventilation [10-12]. Gilbert et al. estimated total per-patient neonatal hospital costs of 202,700 USD for a surviving baby born during the 25^th ^week and 46,400 USD for babies born during the 30^th ^week, decreasing to only 1,100 USD for a 38-week newborn [13].

Besides these studies on the expenses covered by the insurance system, there are also some analyses assessing the parental expenses in this neonatal period: Travel expenses incurred by parents visiting their children in neonatal care units may be considerable if travel to a hospital is entailed. McLoughlin et al. showed that 88% of mothers visited their newborn baby daily and estimated that the median travel expenditure ranged between 101 and 200 GBP [14]. Additionally, there are also other OOPE incurred to families, such as costs related to child care and babysitting for siblings, accommodation expenses during the neonatal hospital stay or lost earn-ings during this time. Referring to this, Tommiska et al. calculated parental mean costs before initial discharge for extremely low birth weight infants (< 1000 g) at 2,755 EUR or 4% of total costs. Travel costs are the main cost category: Travelling induces 64% (1,763 EUR) of all expenses, 30% are earnings losses (827 EUR) and 6% (165 EUR) are payments for accommodation [10]. Gennaro estimated that families spend 2% - 4% of their gross annual income on non-reimbursed out-of-pocket payments, attributable to their infants' condition. OOPE incurred by families of low birth weight infants average 433 USD during the initial hospitalization, with the largest part accounting for transportation (271 USD) [15].

#### Costs after the neonatal period (longer term economic factors)

Most relevant cost components after the neonatal period are expenses for re-hospitalization, outpatient visits, pharmaceuticals, medical aids and non-medical costs for education, travelling, accommodation, child care as well as indirect costs (mainly parental time and/or wage losses). Only few studies have analyzed the long-term economic burden of preterm birth following the initial discharge from the neonatal unit so far. Existing studies are varying with regard to methodological quality, sample size, study design and duration of follow-up [11,16].

As for the short-term costs, post-discharge resource utilization is inversely related with gestational age as well. The majority of costs accrue in the first year of life and costs for re-hospitalization are higher than outpatient costs [9]. For example, McCormick et al. and Stevenson et al. both report that infants born preterm are more likely to incur hospital and other health services (like family practitioner services) during the first years of life than children born at full term and at normal birth weight [17,18]. For the UK Petrou estimated that the adjusted number of hospital inpatient admissions, days and costs over the first 10 years of life was 1.3, 0.77, and 4.43 higher, respectively, for children born before the 28^th ^week. The impact of low gestational age on hospital admissions applied mainly to the first two years of life as opposed to the subsequent period [19].

Most studies do not provide specific disaggregation of the sources of payment for costs incurred by preterm birth, however, some focus on post-discharge parental OOPE and lost productivity. In addition to direct resources consumed, there are also other long-term economic consequences for parents: As a result of additional healthcare contacts during the first years of life, there are remarkable direct non-medical expenses that become a burden on families. An important point are indirect costs: Parents who intended to return to work after the birth often have to reduce their working hours, postpone their return or miss working days to care for their child. Tommiska et al. report parental wage losses of 5,990 EUR in extremely low birth weight infants (< 1,000 g) in the first year of life and 8,175 EUR in the second year - compared to only 880 EUR and 595 EUR for controls, respectively [10].

Travelling costs are estimated at 75 EUR in the first year and at 85 EUR in the second year (compared to 15 EUR in both years for controls). Additionally these authors also report higher OOPE in this group for home aid as well [10]. McCormick et al. have assessed travel costs at 180 USD per year and child care costs at 563 USD [17]. Further substantial costs are special education expenses. Chaikind and Corman, for example, calculated that - compared to children who were of normal birth weight - infants who weighted less than 2,500 g at birth were almost 50% more likely to be enrolled in any type of special education than children who were of nor-mal weight at birth [20]. It should also be noted that there is no data on additional spending as a result of modifications families have to make to cope with everyday activities or payments that have to be made to modify e.g. the home as a result of the infants' impaired health state. Moreover, information on probably needed home help, higher insurance premiums or direct medical co-payments and deductibles is sometimes mentioned, but never estimated in a reliable fashion.

### Quality of life aspects

Health-related quality of life (HRQoL) is an individuals' subjective perception of health status on physical, emotional and social functioning [21]. In pediatric patients this assessment must be seen in the context of the family and interacting influences. On the one hand, the health status of the child has an impact on QoL of the rest of the family, particularly on social and psychological domains. On the other hand, the family situation is influencing the children's well-being very strongly, because the child is dependent on his/her caregiv-ers.

#### QoL of parents

The birth of a premature infant is a critical event in the life of a mother and the rest of the family. A multiplicity of studies tried to describe some of the intangible costs associated with the birth and caring for pre-term and low birth weight children in later life. These studies suggest that the impact is often negative, because of the physical and emotional burden associated with physical illness and the process of caring for the child. Particu-larly the mothers of such infants are at greater risk of psychological distress than mothers of full-term infants [17,22-30].

##### Prenatal and perinatal phase

There have been a wide variety of studies on postpartum depression in mothers of infants born preterm. Depending on study design and included population, there are estimates of between 28% [31] and 70% [22] of preterm mothers as having clinically significant degrees of psychological distress. In a more recent study Davis et al. found that 40% of mothers of preterm infants (< 32 weeks) reported significant depressive symptoms on the Edinburgh Postpartum Depression Scale (EPDS) one month after the birth [23]. This is higher than the population norms of 10% - 15%. Moreover, the estimated percentage is very similar to other studies which have indicated that mothers of premature infants are likely to experience significant depressive symptomatology while their infant is in a neonatal unit [24,32]. During this time parents are negatively influenced by the stress and disappointment of the early birth, self-reproaches, the separation from their fragile child on a neonatal intensive care unit (NICU) with only limited opportunities to interact, the ongoing medical crisis and the possibility of death or continued health and developmental problems of their child [23].

##### Postnatal phase

In the long run (weeks, months and years later) there are also QoL impacts on family members as well: The need for caregivers to provide a high level of vigilance and to maintain this support over months or years may have significant consequences on their own QoL. This may result in maternal depression, self-reproaches, upheaval in the family routine leading to instabilities, marital stress, anxiety of losing the child, emotional and behavioral concerns of siblings, restricted social contacts or sometimes even the feeling of isolation [17,25-30].

#### QoL of infants

There are numerous studies looking at preterm children's HRQoL. As a rule, for preschool-aged children questionnaires such as TAPQOL [33] or Peds-QL [34] were completed by parents. These parent-proxy versions are used, because it can be assumed that children cannot understand the complex theoretical construct of HRQoL in this early age [35]. Sometimes these parent-interviews are used in older ages as well, but normally in school-aged children, adolescents and young adults the HRQoL is self-reported [36]. Most indicate that these persons born preterm are, on average, significantly less healthy (objective QoL) than their normal birth weight peers. They perform more poorly in respect of their physical, emotional and social functioning (e.g. having eating disorders, motor functioning, communicational skills or tend to have problems with anxiety [36,37]). On the other hand the majority of interviewed children or adolescents born preterm do not perceive their own subjective HRQoL significantly different from peers at their age, whereas proxy reports of parents reported significantly poorer performance in their child's global health, behavior and physical functioning. Maybe this difference can be explained by an 'emotional' bias parents might have towards their experience and expectations for their children as well as coping mechanisms by the children over time [26,36,38]. A further possible reason is that severe (or even mild or moderate) problems in early childhood are not present to the children as they forgot them or were too young to memorize.

## Discussion

Economic burden of preterm birth and low birth weight go far beyond the expenses e.g. a health care insurance has to cover as the birth of a preterm child may have an economic impact over many years also on other parties. In fact, this condition imposes a substantial financial burden on the families and caregivers of these infants and then growing children. Despite several studies already targeted costs covered by the insurance system, there is only limited information assessing OOPE, lost productivity and/or impact on QoL for caregivers.

Based on the results of the literature search, a recommendation scheme or a tool box for researchers when constructing a study design, is developed (see table 1). It includes a set of four separate major categories to measure additional healthcare needs and further burden of disease from families' perspective. Each category is divided into several domains covering all relevant aspects of personal burden. For every domain some applicable contents are highlighted, but depending on the respective study design or perspective the selection of individual cost components might differ. Moreover this matrix is not restricted to the application to infants born preterm and/or at low birth weight. These recommendations could be applied to most neonatal diseases like congenital heart defect or RSV infections.

The following components should be considered:

Hospital admissions or re-admissions, increased contacts to general practitioners and other healthcare providers or special requirements on care for everyday living are often connected with individual ex-penses or at least co-payments for the parents. These *direct medical costs *can be divided into outpatient and inpatient cost areas. In addition, *direct non-medical costs *may arise for additional transportation to the hospital and other therapies. Furthermore additional educational needs and care for siblings may have to be arranged. Usually these direct non-medical costs are the most important cost components regarding parental expenditures.

When developing a questionnaire, researchers should define the reported OOPE very precisely to avoid double counting or overlapping between cost categories (particularly between outpatient health care OOPE and other disease-related OOPE, see table 1).

Moreover parents or other caregivers may have to reduce other productive activities, such as paid work, in order to spend more time with their children. These lost earnings, missed working days and time losses by parents or other caregivers who are unable or less able to work represent *indirect costs *[11].

Other problems subsequently may arise through *intangible costs*, in terms of reduced QoL of parents. The instruments which can be used to evaluate caregivers' QoL or distress level can roughly be divided into instruments for the acute hospitalization period (regarding mainly feelings of the mother) and those for continued evaluation in later months or years for the whole family. Some examples for frequently used instruments in studies evaluating feelings of parents with preterm infants are:

### Impact on parents during neonatal period (infants' hospitalization)

- Parental Stressor Scale: Neonatal Intensive Care Unit (PSS:NICU): This questionnaire measures parental perception of stressors arising from the physical and psychosocial environment of the NICU due to alterations in the parental role, staff relationships, infant behavior and appearance, and unit sights and sounds. There is both an interview and a self-reported format available. The instrument has shown evidence of internal consistency, reliability, and validity [39].

- Edinburgh Postpartum Depression Scale (EPDS): This 10-item self-report scale is a well-validated and widely used screening tool for depression after birth of a child. The questionnaire focuses on the cognitive and affective features of depression rather than somatic symptoms. The EPDS is not diagnostic, but gives an estimate of psychological disturbance and alerts health care professionals that further assessment is required. The score is ranging from 0 to 30, whereas a cut-off level of 12 has been used in several studies to indicate probable depressive disorder [40].

- Spielberger State-Trait Anxiety Inventory (STAI): A self-report instrument designed to measure and to differentiate between anxiety as a state and a trait. It assesses overall stress reactions (state anxiety: 20 items) and personal stress traits (trait anxiety: 20 items). The measure has been used previously to assess parental anxiety with a child's hospitalization [41].

### Impact on the family in following months and years

- Impact on Family Scale: This scale measures the impact that a child's illness has on family function. Financial burden, familial/social impact, personal strains, and mastery abilities are assessed to subscales that can be summed to a total score as well. Reliability and validity in evaluations with premature or low birth weight infants are proven in former studies [42].

- Family Adaptability and Cohesion Evaluation Scale II (FACES II): This 30-item measure evaluates parental perceptions of family adaptability and cohesion. Higher scores on the adaptability subscale indicate higher flexibility, whereas higher scores on the cohesion subscale indicate better emotional family connection [43].

In addition to these generic scales without any disease-specific background, there is a recently developed questionnaire assessing the impact on parents of an infants' hospitalization for bronchiolitis. This disease usually affects infants of less than two years, particularly following preterm birth. The Impact of Bronchiolitis Hospitalization Questionnaire (IBHQ) contains 65 items, which were organized into 8 sections: parent emotional impact, infants' reactions, parent physical reactions, impact on daily organization, siblings' reactions, parent behavior with infant and siblings, impact on couple, and financial consequences [44]. Of course, not every premature infant is hospitalized for bronchiolitis, but these dimensions might also be adequate to measure the impact on families in case of prematurity in general.

Besides the instruments regarding parental well-being, there are some studies assessing the QoL of preterm children as well. Depending on the age, there are several instruments questioning the child directly, whereas others prefer to use a parent as a proxy respondent. It is estimated that children can begin reporting more complex domains of their own HRQoL between the age of 4 and 6 years [35]. In very young children and preschoolers, judgment about their own QoL is only possible by parent-proxy reporting. Although this is necessary, parental ratings are influenced by their own feelings towards and expectations for their children. For school-age children and adolescents instruments questioning directly should be preferred [36,45].

One frequently used proxy instrument is the 43-item TNO-AZL Preschool Children Quality of Life Questionnaire (TAPQOL) as a generic instrument for assessing HRQoL of preschool children age 1 to 5 years. It is consisting of 12 multi-item scales that cover the domains physical, social, cognitive, and emotional functioning. It is commonly used for research among preterm infants and after hospitalization for RSV [33]. Other generic instruments regularly used are Pediatric Quality of Life Inventory (Peds-QL) [34], Health Utility Index II (HUI2) [46] or Child Health Questionnaire (CHQ) [47]. In later life the usage of more global generic instruments like EQ-5D [48] or SF-36 [49] is possible as well.

It is to emphasize that at least the direct medical and non-medical cost dimensions should be included in any health economic evaluation regarding neonatal problems. The additional consideration of indirect costs and QoL aspects allows an even more detailed and comprehensive view on the total burden from the families' perspective. Deviations may occur due to different views but should be justified with a detailed description of chosen study design. A more standardized assessment according to these suggestions would be an important step to a higher level of comparability of results from different countries. On the contrary, in particular OOPE are highly depended on legal framework. Parental payments may vary widely between different health care systems so that an unadjusted transfer of results is not appropriate.

Empirical studies always pose challenges with regard to recall bias and selection of the survey group. It is difficult to verify the reliability and validity of the responses. However, findings should be based on a large cohort of parents in a geographically defined area and include a comprehensive record of out-of-pocket pay-ments as well as other fields. Reports should always be checked for face validity and internal logic [50]. Ideally information on in- and outpatient visits, medication or aids should be confirmed directly with the health insurance or the physicians' office. On the contrary, reported OOPE, changes of income or QoL variations are difficult to verify. The identification of cost attributable to the preterm birth, in comparison to costs which would have been induced by a normal term birth as well, would be possible by the inclusion of a control group (infants born at term), especially considering that it is difficult for parents to separate these costs in an interview.

A preferable way to evaluate the economic and emotional burden of a preterm birth on the family is to ask combined closed- and open-ended-questions about medical and non-medical services not covered by their health plan and paid for by the parents (OOPE, co-payments, and deductibles). These information as well as missed working days, reduced income, lost time and especially subjective rating of own stress level or QoL are ideally assessed from the parents themselves.

Potential questions regarding the different cost dimensions should be tested with some adequate respondents to verify their completeness and relevance. In this pretest participants should be encouraged to give a detailed answer and to speak about their experiences. Thereby researchers' assumptions about the economic and social burden of disease are reassessed [21].

Ideally the questions would not be limited to the parents' own experiences so that burden on other persons concerned was evaluated as well. However, the integration of multiple caregivers could potentially lead to confusion, most notably if parents were asked to estimate OOPE or time spent by other volunteers (e.g. additional, non-paid childcare for other siblings). This is why the collected information should be restricted to burden on the main caregiver (usually parents), keeping in mind that this limitation could potentially underestimate total societal burden, because other persons affected are not included [51].

Generally, a questionnaire is only able to capture a limited period of time. To minimize negative effects, parental burden should be derived from very detailed and separated questions (e.g. travelling distances, medication, therapies etc.). Parents are only able to give valid information on their own expenses for a limited de-fined period. For example, information on parental transportation and accommodation costs should be evaluated directly on the day of discharge or, even better, daily during the hospitalization. Long-term non-reimbursed costs of ongoing medical and non-medical needs provided by families, additional loss of income or an enduring feeling of stress and anxiety should be evaluated by separated and regularly repeated interviews. Primarily cost-calculations for later life would be very interesting; in the literature there is only scarce evidence as to whether costs remain higher in future life. A long-term longitudinal study, with parents surveyed periodically over several years, would be valuable to follow the development of preborn infants. Realizing this, there is a need for specific questionnaires representing different periods of life (infant vs. later childhood vs. adolescence). For example, educational costs or payments for therapies like logopedics are only relevant in later life and not in the first weeks.

It also should be discussed, what form of questionnaire is really adequate for the evaluation of complex and detailed economic burden, particularly if a QoL questionnaire for parents and/or children is included in the study [26,36,37]. For measuring QoL or the long-term consequences with regard to a changed income situa-tion, an evaluation only once a month or maybe just several times per year may be sufficient. Particularly QoL aspects should not be measured via internet, because most of the instruments are only validated for a written (or sometimes an oral) survey.

To address certain limitations and to gain a deeper insight into this topic additional research should be conducted in the future. Developed recommendations need to be discussed and tested: this includes research on the feasibility of included dimensions as well as validation of parent-reported information on burden. Moreover, the sample size of future studies or surveys should be big enough in order to yield several subgroups within the study sample. This could be combined with an international study incorporating more countries in the survey. It would be most interesting to explore whether there are diverging results in different health care systems and to explore the reasons and resulting incentives.

Future research should also try to identify cost-effective interventions which have the potential to prevent preterm births and reduce morbidity and mortality of infants and mothers once a preterm birth occurs. It is important that the economic impact (including parental OOPE, productivity losses as well as QoL impact of prematurity and connected diseases such as RSV on infants and their families) is recognized in future studies evaluating pre-, peri- and neonatal treatment strategies. To identify an efficient allocation of resources, data on incremental costs and health benefits attributable to particular interventions are needed [19]. RSV infections could be chosen as an example at this point, because they are a prevalent and more or less well investigated health problem associated with prematurity. They are a good example for an area where health economic evaluations already exist, but investigators frequently neglect to include the family perspective when calculating burden. With a full health economic evaluation - including total burden on families - it could be analyzed whether preventing or treating infections in the at-risk infants offers a cost-effective approach in order to reduce costs on society and parents.

## Conclusions

In conclusion, this study has illustrated the large extent of burden on parents of a child born pre-term. There is a significant (economic) burden on the families besides the medical burden and further financial burden to the health insurance system. It was shown that preterm birth and low birth weight result in substantial OOPE and emotional distress to the parents. The largest part of parental burden occurs in the long run after infants are discharged from the neonatal unit. Nevertheless emotional stress is on a very high level during initial hospitalization and significantly increases with decreasing gestational age.

Economic evaluations are performed rather inhomogeneously in this field and seemingly were a rather neglected topic in recent years. Generalized recommendations including all important domains to measure financial and emotional burden on families have been developed. Further research should follow these methodological introductions to get a more detailed, comprehensive, standardized and comparable view from the families' perspective.

It is important that decision-makers, health-insurers and healthcare providers are aware of the total clinical, financial and emotional burden borne by parents at this critical time in the parent-child relationship. At this moment evidence is missing to convince decision makers of the seriousness of parents' perspective. The existence of comprehensive information on total costs to society (including parental burden) would help to make decisions on a broader basis. Considering different sources of expenditures and personal distress could lead to decisions targeted on a reduction of total societal burden and not on health insurance burden alone.

## Competing interests

Research work was supported by Abbott Laboratories.

## Authors' contributions

JMH, JMS and TM all participated in the design, concept and draft of the review. JMH and TM performed the writing. All authors read and approved the final manuscript.
